# Developmental photoperiod alters the onset and capacity for reproduction in male dark-eyed juncos (*Junco hyemalis*)

**DOI:** 10.1098/rspb.2026.1696

**Published:** 2026-10-14

**Authors:** Carlie G. Saline, Adam M. Fudicker, Allison J. Byrd, Ellen D. Ketterson, Timothy J. Greives

**Affiliations:** ^1^ Biological Sciences Department, North Dakota State University, Fargo, ND 58105, USA; ^2^ Center for the Integrative Study of Animal Behavior, Indiana University Bloomington, Bloomington, IN 47405, USA; ^3^ Department of Biology, Indiana University Bloomington, Bloomington, IN 47405, USA; ^4^ Tin Mountain Conservation Center, Albany, NH 03818, USA

**Keywords:** developmental plasticity, photoperiod, seasonal timing, reproduction, hypothalamic–pituitary–gonadal axis, songbird, cloacal protuberance, phenology

## Abstract

Natural selection favours individuals able to physiologically adjust to seasonal environmental cues, such as photoperiod, and time their reproduction accordingly. Although it is known that differences along the hypothalamic–pituitary–gonadal (HPG) axis can explain differences in reproductive timing across the dark-eyed junco *(Junco hyemalis)* range, it remains unknown whether these differences arise from local adaptation or early-life environmental effects. We exposed nestlings from a single population to photoperiods mimicking development at the northern and southern extents of the species’ breeding range. In autumn, we adjusted the photoperiod in the northern treatment to mimic southward migration, after which all birds experienced the same daylengths through the winter to simulate mixed overwintering flocks in the wild. Beginning early March, birds were injected with gonadotropin-releasing hormone (GnRH) to measure induced testosterone as a proxy for HPG axis maturation; brood patches, cloacal protuberances and fat were also measured. Males raised under northern developmental photoperiods delayed the onset of reproductive readiness compared with control males. Sample date affected females’ GnRH-induced testosterone and fat deposition but not in clear patterns. These results indicate the daylength under which male songbirds develop can influence their reproductive schedules. Further research should discern the interplay of plasticity and genotypes and potential sex-specific differences.

## 1. Introduction

Within temperate zones, it is evolutionarily favourable to raise offspring in seasons of amenable climate and abundant resources [1,2]. Although temperature and other factors can provide proximate cues to fine-tune the precise timing of reproduction, photoperiod is the main predictive seasonal cue by which animals align their reproductive status with environmental suitability [2–6].

In vertebrates with relatively short gestation or incubation periods (i.e. long-day breeders, such as songbirds and rodents), lengthening photoperiod triggers the hypothalamus to release gonadotropin-releasing hormone (GnRH), which travels via the hypophyseal portal system to the anterior pituitary. Here, the decapeptide activates gonadotropes to release luteinizing hormone (LH) and follicle-stimulating hormone (FSH). These glycoproteins act in concert to stimulate sex steroid production and gametogenesis in the gonads, while upstream receptors modulate steroid levels via negative feedback [7].

Within species, populations often vary in reproductive timing across their ranges to correspond with local variation in climate and resource availability (mice [3], turtles [8], anurans [9]). In birds, Silverin *et al.* [10] observed that great tits (*Parus major*) captured in Gothenburg, Sweden (57.7° N latitude) required a longer photoperiod (that is demonstrated a higher photoperiod threshold) before testicular recrudescence compared with individuals captured at lower latitudes in Milano, Italy (45.4°N latitude). Additionally, males originating from Gothenburg maintain recrudescent testes for a shorter amount of time in the summer compared with Italian males, reflective of the shorter breeding season further north [10].

More recently, a study of migrant and resident dark-eyed juncos (*Junco hyemalis* spp.) captured on their shared overwintering grounds in Virginia, USA noted that in captivity, migrants originating from further north (based on deuterium values in feathers) also require a longer photoperiod to activate seasonal reproductive physiology, as indicated by testis mass, cloacal protuberance (CP) volume and baseline testosterone ([11], see also [12] for a free-living comparison). These observations suggest that local adaptation can pair reproductive timing with local resources (reviewed in [13]).

Indeed, such variation in photoperiodic thresholds across species’ ranges has been historically attributed to local adaptation, implying a genetic basis [10,13–17]. Species’ coordination of physiological and behavioural responses with seasonal rhythms is often interpreted as adaptive specialization (traditional local adaptation) or conditional plasticity (differential utilization of a ‘jack-of-all-trades’ genome throughout adulthood) [18]. However, such a framework, largely based on studies with wild-caught adults, does not eliminate potential effects of the natal environment (i.e. developmental plasticity). The relative importance of these forces—local adaptation, the ability to plasticly respond in adulthood and developmental plasticity—may weigh differently in the shaping of seasonal timing among populations.

Regarding developmental plasticity, recent work in mice demonstrates that the photoperiod an individual experiences early in life can influence its circadian rhythms into adulthood [19]. Given that circadian rhythms influence seasonal rhythms of reproduction via light detection (photostimulation) during receptive periods of the circadian cycle (photosensitive phases), such effects of developmental plasticity, perhaps in conjunction with local adaptation or plasticity in adulthood, may explain the variation in reproductive timing and photoperiodic thresholds observed across species ranges [4,10,11,20,21].

We experimentally tested the hypothesis that developmental photoperiod impacts the establishment of the seasonal photoperiodic response system, and therefore the timing of the seasonal reproductive transition, with a well-studied songbird system, the dark-eyed junco*.* This species’ range spans a large latitudinal cline such that populations display substantial variation in the timing of breeding [12,22,23]. Across North America, populations further north exhibit higher photoperiodic thresholds (and therefore delayed, shorter breeding seasons), while those further south exhibit lower photoperiodic thresholds (and earlier-onset, longer breeding seasons) that align with their environments [11,13]. Even when these populations overwinter together, they retain their different reproductive schedules, as in the ‘sympatric but allochronic’ community in the Appalachian Mountains of Virginia [22,24,25].

Our hypothesis builds on the common-garden study of Fudickar *et al.* [24], in which wild adult migrant and resident male juncos were exposed to increasing daylength in captivity. Residents secreted more testosterone in response to GnRH injections and grew larger CPs (the sperm transfer organ) earlier in spring compared with migrants (see also [11]).

To investigate whether seasonal reproductive timing is sensitive to the photoperiod experienced during development through their first autumn, we exposed recently hatched nestlings from a single breeding population *(J. h. carolinensis* in Virginia, USA) to photoperiods they would have experienced had they developed at a higher latitude (‘northern-treated’) or the lower latitude (‘southern-treated’) of their actual hatch site in Virginia, spanning the species’ breeding range [23]. All birds experienced the same photoperiod over the winter, mimicking their shared environment at our study site in Virginia.

If developmental photoperiod influences the timing of reproductive development, we predicted that early in the following spring, while still experiencing identical photoperiods, birds that developed under the northern photoperiod would delay reproductive readiness via reduced GnRH-induced testosterone and smaller CPs or brood patches (BPs). We predicted that birds developing under photoperiods mimicking the southern, control photoperiod would display earlier growth of CPs and BPs and greater testosterone secretion in response to GnRH, indicating an active hypothalamic–pituitary–gonadal (HPG) axis and preparation for breeding.

## 2. Methods

### (a) Experimental design

From 12 May to 4 June 2021, nestlings found within 3 km of Mountain Lake Biological Station (Pembroke, Virginia) were sexed by collecting buccal swabs on hatch day and amplifying the chromodomain helicase DNA binding (*CHD*) gene on the W and Z chromosomes via polymerase chain reaction; the presence or absence of the W chromosome indicated female or male, respectively [26,27].

Approximately 2 days post-hatch, nestlings were brought into captivity and assigned to treatments such that broods were separated and sexes balanced between treatments. The treatments comprised a control ‘southern photoperiod’ with the same light-dark schedule birds would have experienced had they remained in their nests (based on Blacksburg, Virginia (37.2296°N, 80.4139°W)), and an experimental ‘northern photoperiod’ (based on McGrath, Alaska (62.9532°N, 155.5960°W)), which mimicked the daylengths juncos experience at the northern extent of their breeding range [23,27]. Artificial sunrise and sunset times were updated every other day.

Birds were fed mealworms every 30 min from sunrise to sunset until individuals no longer gaped for food. Birds in both treatments were housed in incubators maintained at 29.5°C and 60% relative humidity until they were 7 days old. At that point, they were moved to incubators maintained at 24.5°C and 60% relative humidity. When birds were about 12 days old, the age at which juncos typically fledge, birds were moved from incubators to cages in a room maintained at 22°C (for further detail on molecular sexing and captive conditions, see [27]).

On 27 June 2021—when all individuals were at least 23 days old and, therefore, past the age at which they would have fledged in the wild—birds were transported from Mountain Lake Biological Station to Indiana University (Bloomington, Indiana, USA) by vehicle in large individual paper bags. Birds were divided by treatment into two indoor aviaries (dimensions 2.5 m long, 2.1 m wide and 2.4 m high) to allow them the required space in captive conditions. Groups resumed experiencing their respective daylengths in their separate aviaries.

From 13 September to 22 October, northern-treated nestlings experienced changes in daylength that mimicked what they would have experienced during a southward migration until both treatments experienced the same control/southern photoperiod, simulating the populations’ sympatry through winter and into early spring. On 24 February 2022, birds were individually caged with blinders between adjacent cages to facilitate recording of individuals’ measurements. The use of two rooms was necessary owing to size constraints, but room assignment was random, and treatment and sex were balanced between the two rooms. See figure 1 for a schematic of daylengths experienced by treatments during the study.

**Figure 1.**
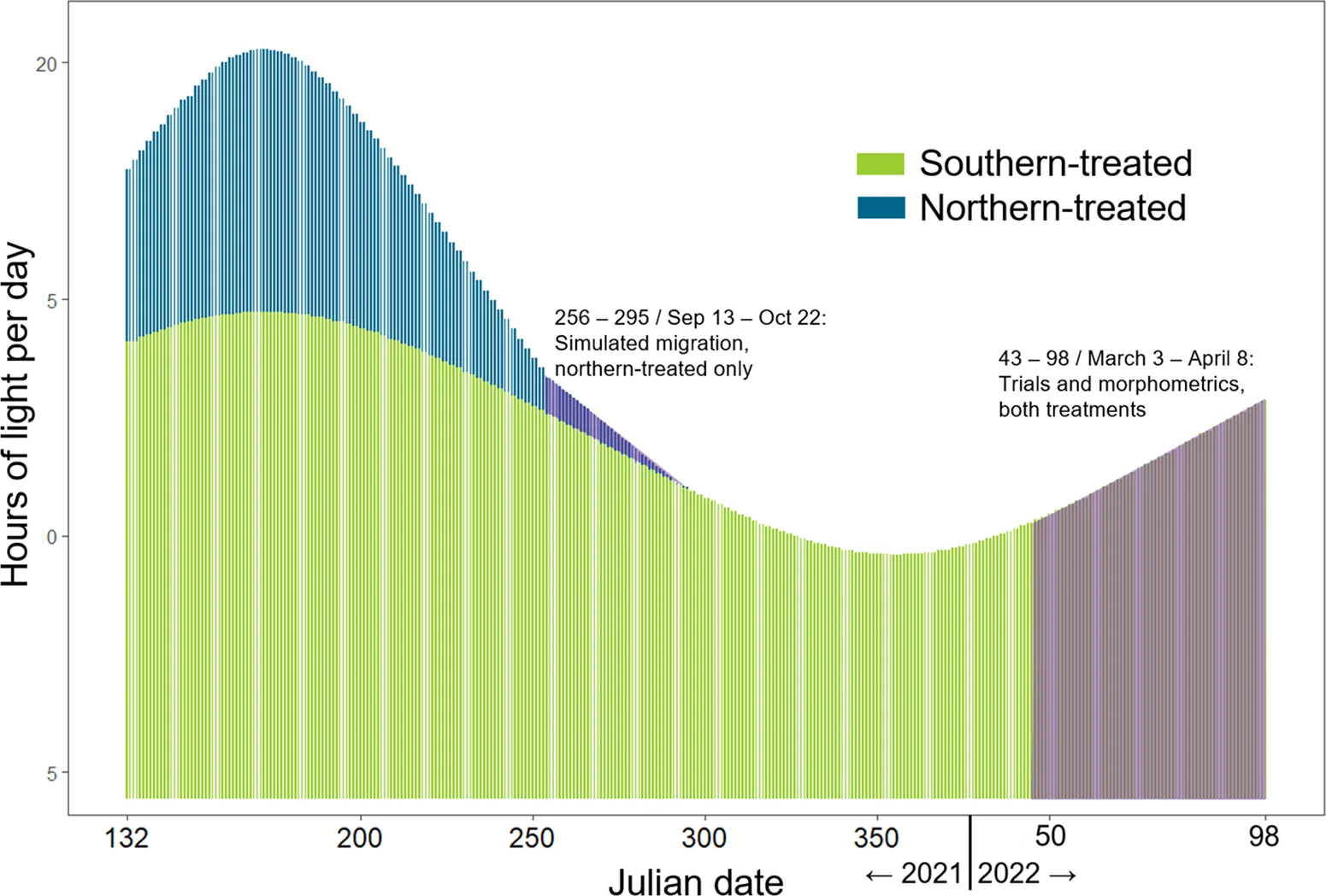
Schematic of photoperiods experienced throughout the experiment. Nestlings were brought into captivity and assigned treatments for Julian dates 132–155 (12–15 May) 2021. From Julian dates 256–295 (13 September–22 October), northern-treated individuals’ daylengths increased to simulate a southward migration (shaded triangle), such that both groups experienced the same amounts of daylight for the rest of the experiment to mimic sympatric overwintering. Hormone trials and morphometric assessments (shaded quadrilateral) ran from Julian dates 43–98 (4 March–8 April) 2022.

Beginning 5 March 2022, we assessed the capacity for testosterone production in response to a standardized weekly injection of 1.25 µg chicken GnRH (Bachem AG, Bubendorf, Switzerland) in 50 µl of phosphate-buffered saline into the pectoralis muscle. Thirty minutes later, the alar vein in the wing was punctured and approximately 70–110 µl blood collected to measure the HPG axis’ capacity to respond and release testosterone [28]. To minimize unnecessary blood loss, we did not record baseline testosterone.

Mass was recorded to the nearest 0.01 g; furcular and abdominal fat were scored separately from 0–5 based on the scale established by Nolan & Ketterson [29] and then summed. Note that one northern-treated male's fat scores were not recorded the last week of the experiment, and one northern-treated female's and one southern-treated female's fat scores were not recorded for weeks 2 and 3, respectively. The same researcher quantified all fat scores to further standardize this measurement. Female BP was scored from 0–5 based on the British Trust for Ornithology scale, and male CP width and height were recorded with calipers to the nearest 0.1 cm. CP volume was then estimated with the equation for the volume of a cylinder [30].

### (b) Lab methods

We measured testosterone with enzyme-linked immunosorbent assay (ELISA) kits for males (kit number ADI-901-065, minimum detection 5.67 pg ml^−1^ (range 7.81–2000 pg ml^−1^), species independent) or high sensitivity testosterone ELISA kits for females (kit number ADI-901-176, minimum detection 2.6 pg ml^−1^ (range 3.9–1000 pg ml^−1^), human) (Enzo Life Sciences, Farmingdale, NY, USA).

We measured testosterone to assess HPG axis maturation in females because the plasma volume required for our validated oestradiol assay (approximately 100 µl) was too high to safely collect repeated samples every week [31]. We instead measured testosterone, which requires significantly less plasma (approximately 30 µl) and is the substrate that aromatase converts into oestrogen. Prior work in our group has demonstrated that post-GnRH testosterone and oestradiol levels in females are correlated and that resident females secrete more testosterone 30 min after GnRH injection compared with migrants during winter sympatry [32,33].

After approximately balancing the representation of sample dates and treatments, samples were randomly distributed on plates within sexes (males = four plates, females = four plates). All samples were extracted with ether twice. Twenty microlitres of plasma was used in the male assays and 30 µl in female assays. Although some samples from females were less than 30 µl, the average volume was 28.5 µl. No samples for males or females were below the minimum detection limits. The intra- and inter-plate coefficients of variation for females were 9.55% ± 2.56% and 5.25% ± 0.48%, respectively. The intra-and inter-plate coefficients of variation in males were 6.12% ± 1.22% and 8.77% ± 0.26%, respectively.

### (c) Statistical methods

Normality was assessed in all datasets with the Anderson–Darling test and log-transformed where appropriate to improve normality. Fat scores and GnRH-induced testosterone, while not normally distributed, were closest to normal when raw values were used as opposed to transformed values. GnRH-induced testosterone, CP volume, and sums of furcular and abdominal fat scores were analysed separately with linear mixed models via the function lmer from the package lmerTest [34]. Fixed effects included treatment, sample date, nest ID (as a proxy for relatedness) and the interaction of treatment and sample date, while individual bird was a random effect with the lme4 package in R (version 4.2.1, R Foundation for Statistical Computing, Vienna, Austria [35]). We conducted Tukey's pairwise comparison to investigate significant effects with the function lsmeans from the package emmeans [36]. In females, GnRH-induced testosterone and summed fat were analysed with the same models as for males. We did not analyse BP as no females in either treatment exhibited any BP development during the 6-week sampling period.

## 3. Results

### (a) Males

No main effect of developmental photoperiod or nest of origin on testosterone secretion in response to GnRH injection was detected (figure 2, *n* = 26, treatment: *F*_1,8_ = 0.45, *p* = 0.52; nest ID: *F*_16,8_ = 0.76, *p* = 0.70). However, we found a significant main effect of sampling date (*F*_5,120_ = 15.74, *p* < 0.01) and a significant treatment-by-week interaction on GnRH-induced testosterone (*F*_5,120_ = 3.65, *p* < 0.01). Southern-treated males secreted more testosterone than northern-treated males in early and mid-March (*n* = 13 per treatment, *p* < 0.05), after which there was no difference in testosterone.

**Figure 2.**
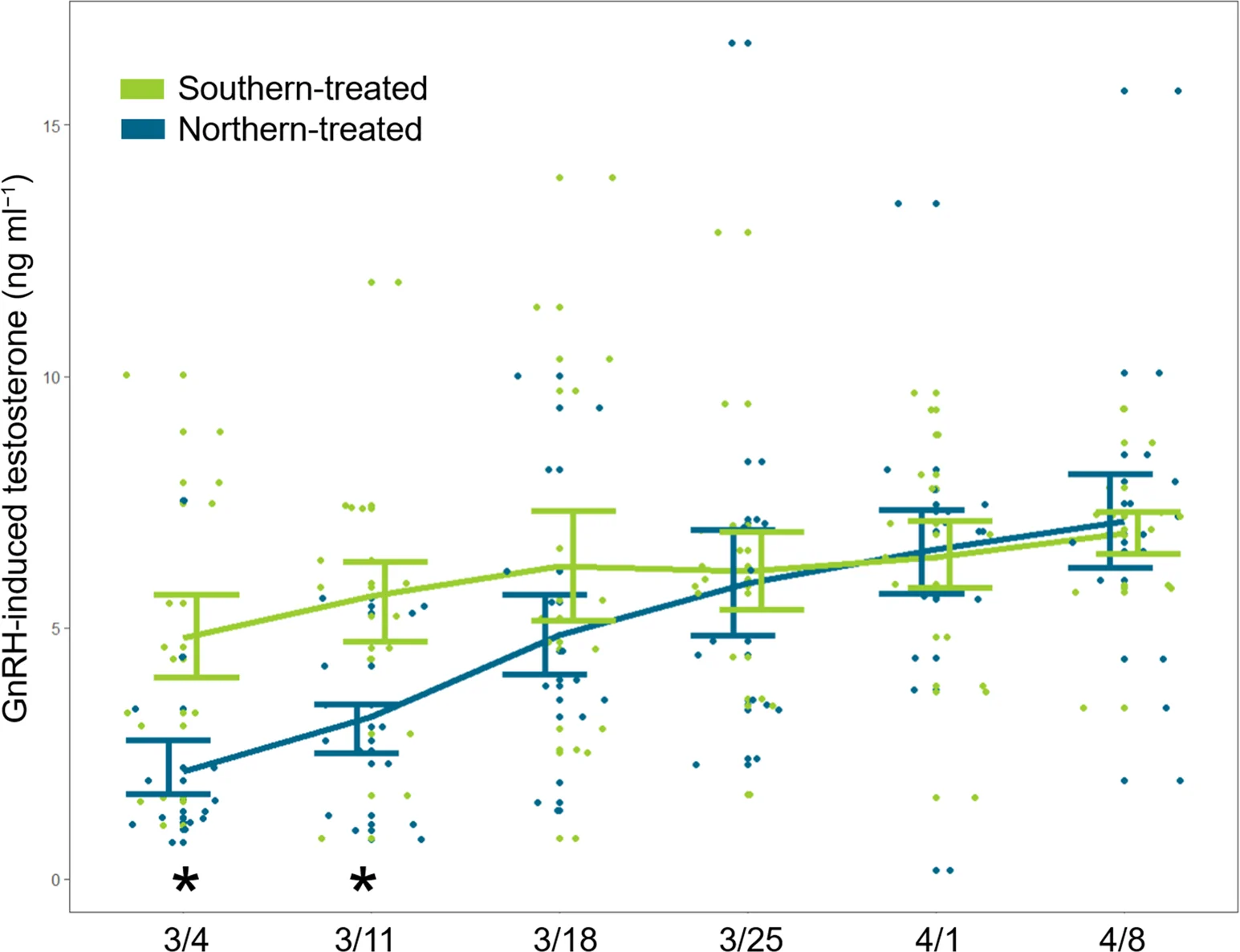
GnRH-induced testosterone (ng ml^−1^, mean ± s.e.m.) in males. There was a main effect of sampling week, and the interaction of sampling week and treatment was significant (*p* < 0.05), such that southern-treated males exceeded northern-treated males (*n* = 13 per treatment) during the first 2 weeks of March (3/4 and 3/11). Asterisk denotes pairwise comparisons with *p* < 0.05.

Though we detected no significant main effect of treatment for log-transformed CP, southern-treated males averaged greater raw-value CP volumes (figure 3, log-transformed effect of treatment: *n* = 26, *F*_1,8_ = 0.29, *p* = 0.60; final values (mean ± s.e.m.): 157.63 ± 6.17 mm^3^ in southern-treated males versus 116.60 ± 5.84 mm^3^ in northern-treated males). A main effect of sampling week was observed on log-transformed CP volume (*F*_5,120_ = 28.23, *p* < 0.01), such that the average value on 4 March was smaller than the averages of 18 March–8 April (*p*’s ≤ 0.05). Similarly, the average value of 11 March differed from all weeks thereafter, as did the average of 18 March with all following dates (*p*’s < 0.05). We did not observe a treatment-by-week interaction or effect of nest ID on CP volume (treatment-by-week interaction: *F*_5,120_ = 0.93, *p* > 0.05; nest ID: *F*_16,8_ = 1.34, *p* > 0.05). However, southern-treated males tended to have larger CPs during the last 3 weeks of the experiment (figure 3).

**Figure 3.**
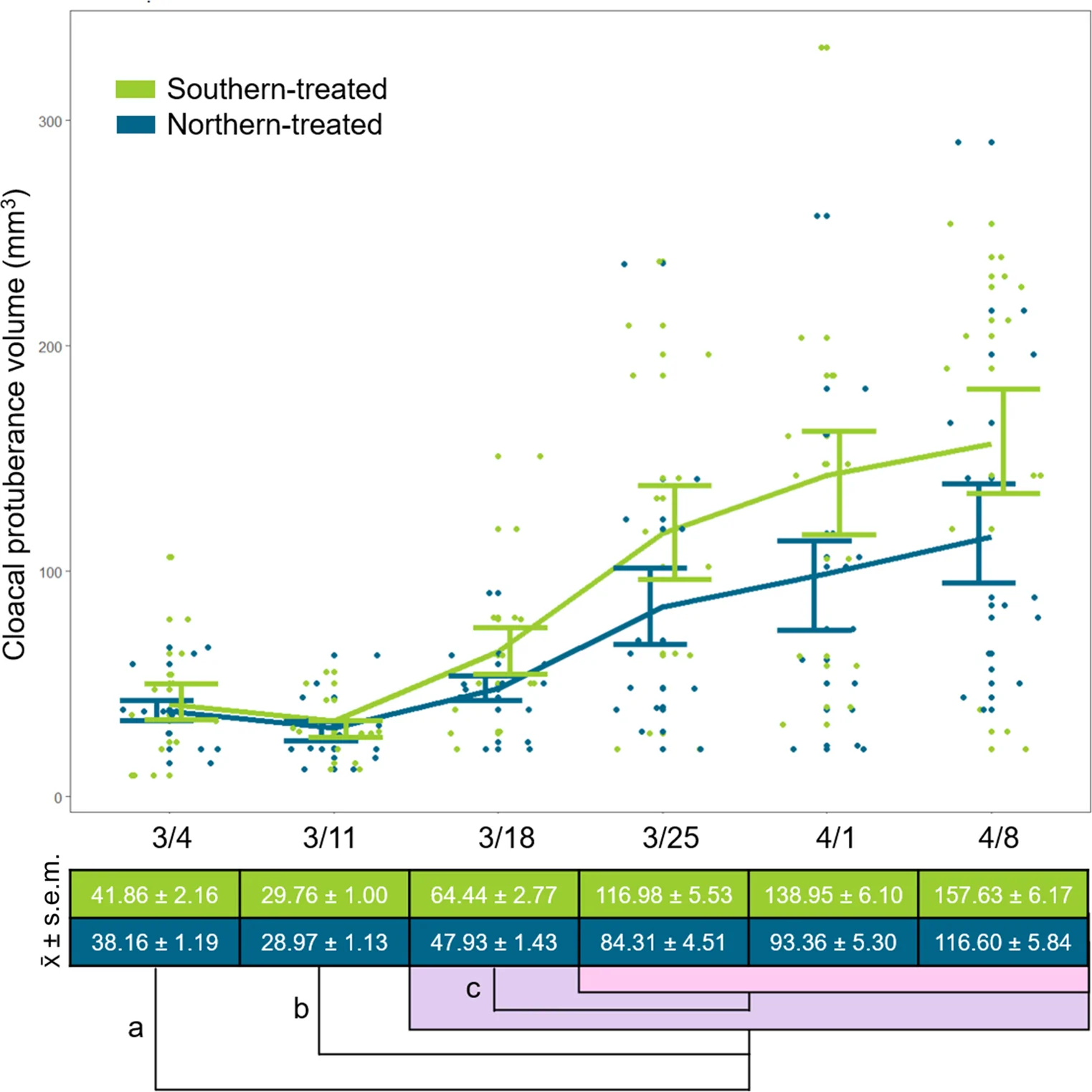
Raw CP volume (mm^3^, mean ± s.e.m.) in males. No significant differences were observed in the 6 weeks prior to the end of the experiment, though the CPs of southern-treated males were consistently larger than those of northern-treated males (*n* = 13 per treatment). A main effect of sampling week was observed on log-transformed CP volumes such that 4 March differed from 18 March–8 April (letter a), 11 March differed from 18 March–8 April (letter b) and 18 March differed from 25 March–8 April (letter c) (*p*’s < 0.05). Note that raw values are shown in the table for simplicity.

We did not observe significant effects of treatment, sampling date, their interaction or nest of origin on summed fat in males (figure 4, *n* = 26, *p*’s > 0.05). While not significant, average fat scores were greater in northern-treated males (*n* = 13 per treatment, northern-treated summed: x̄ = 6.21 ± 0.34; southern-treated summed fat: x̄ = 5.04 ± 0.26).

**Figure 4.**
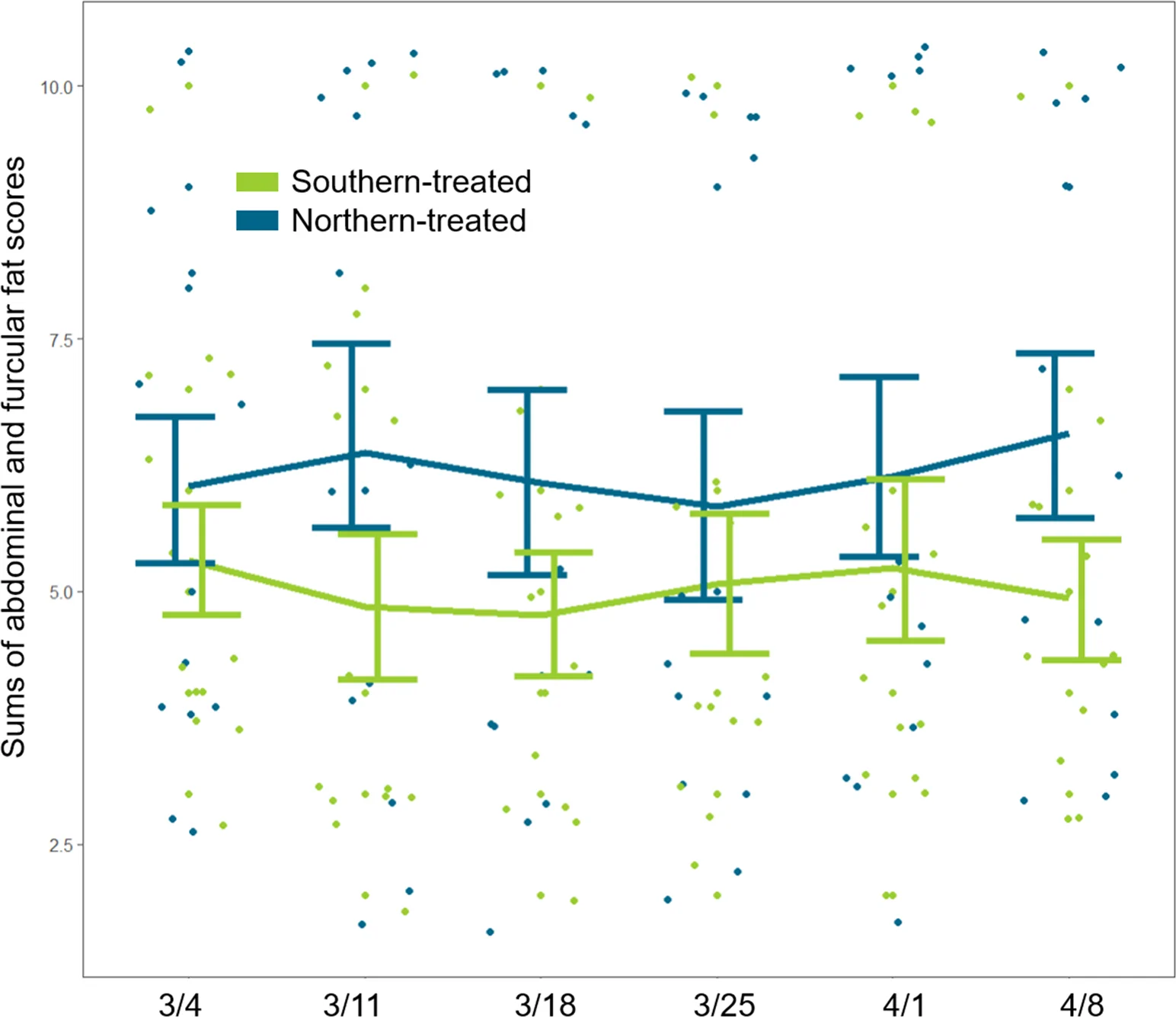
Sums of furcular and abdominal fat scores in males (mean ± s.e.m.). No significant differences were observed in the 6 weeks prior to the end of the experiment, though northern-treated males scored consistently higher than southern-treated males (*n* = 13 per treatment). Note that while models were run with log-transformed values, raw values are graphed for ease of viewing.

### (b) Females

Testosterone in response to GnRH injection did not vary by developmental photoperiod treatment, nor did we observe a treatment-by-sampling week interaction or effect of nest of origin (*p*’s > 0.05). There was, however, a significant main effect of sample date on GnRH-induced testosterone (figure 5, *n* = 22, *F*_5,97_ = 3.17, *p* = 0.01). On average, we observed maximum GnRH-induced testosterone concentrations in early March before dropping again to the lower levels seen at the beginning of the trials. Average circulating testosterone was highest on 11 March and significantly higher than the averages for 4 March and 1 April (4 March versus 11 March: *t*_97_ = −2.97, *p* = 0.04; 11 March versus 1 April: *t*_97_ = 3.05, *p* = 0.03). Average testosterone on 11 March trended towards being significantly greater than that of 8 April (*t*_97_ = 2.82, *p* = 0.06).

**Figure 5.**
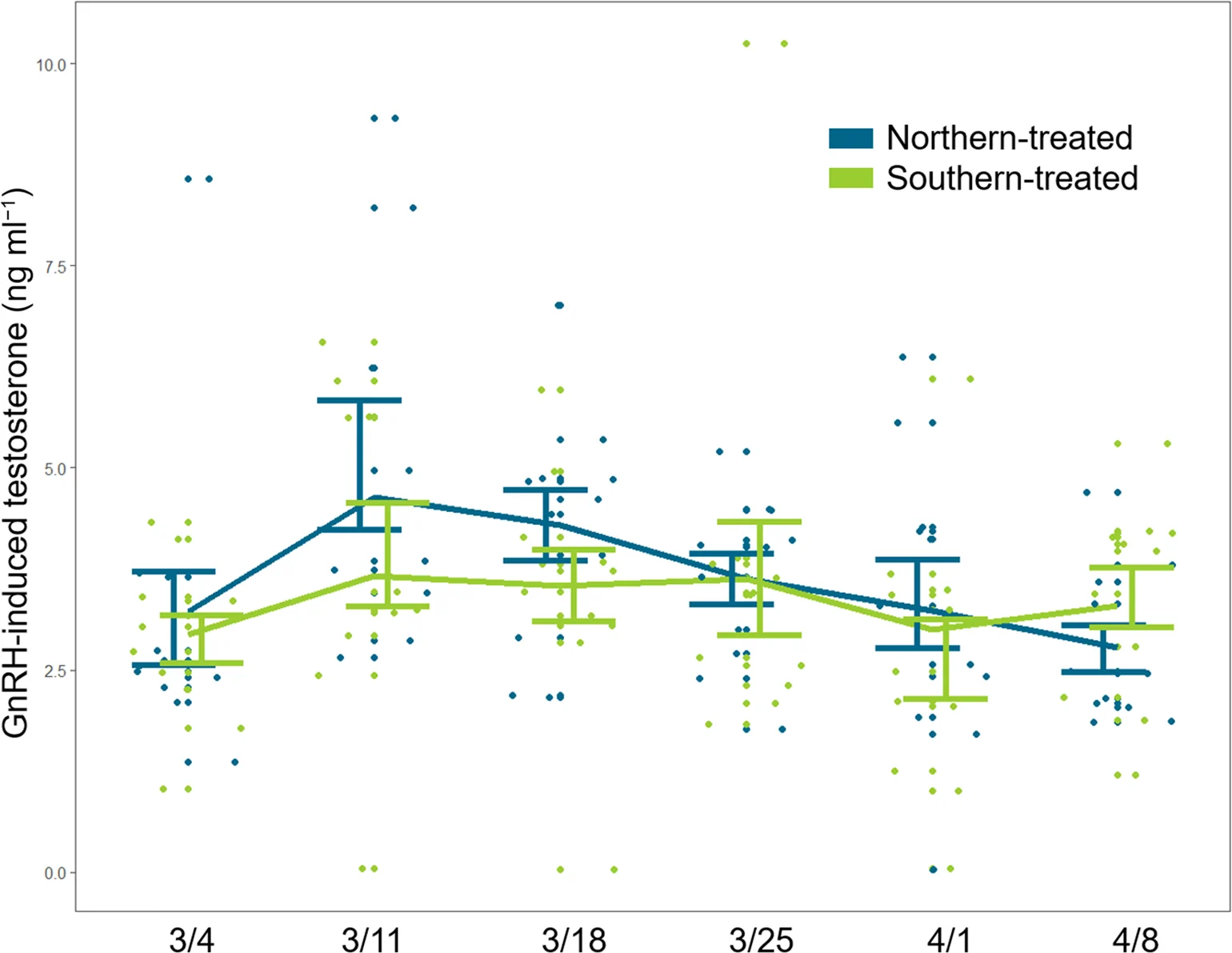
GnRH-induced testosterone (ng ml^−1^, mean ± s.e.m.) in females (*n* = 11 per treatment). No differences were observed between treatments.

Developmental photoperiod treatment had no effect on females’ BPs, which remained at the lowest ranking in both groups for the duration of the experiment.

Sampling date and nest of origin influenced female fat deposition (figure 6, week: *F*_5,98_ = 2.62, *p* < 0.05; *F*_14,6.10_ = 5.63, *p* < 0.05); however, no significant *post hoc* pairwise differences were identified. Developmental photoperiod and its interaction with sample date did not significantly influence female fat deposition (treatment: *F*_1,6_ = 2.30, *p* = 0.18; treatment-by-week: *F*_5,98_ = 0.24, *p* = 0.94).

**Figure 6.**
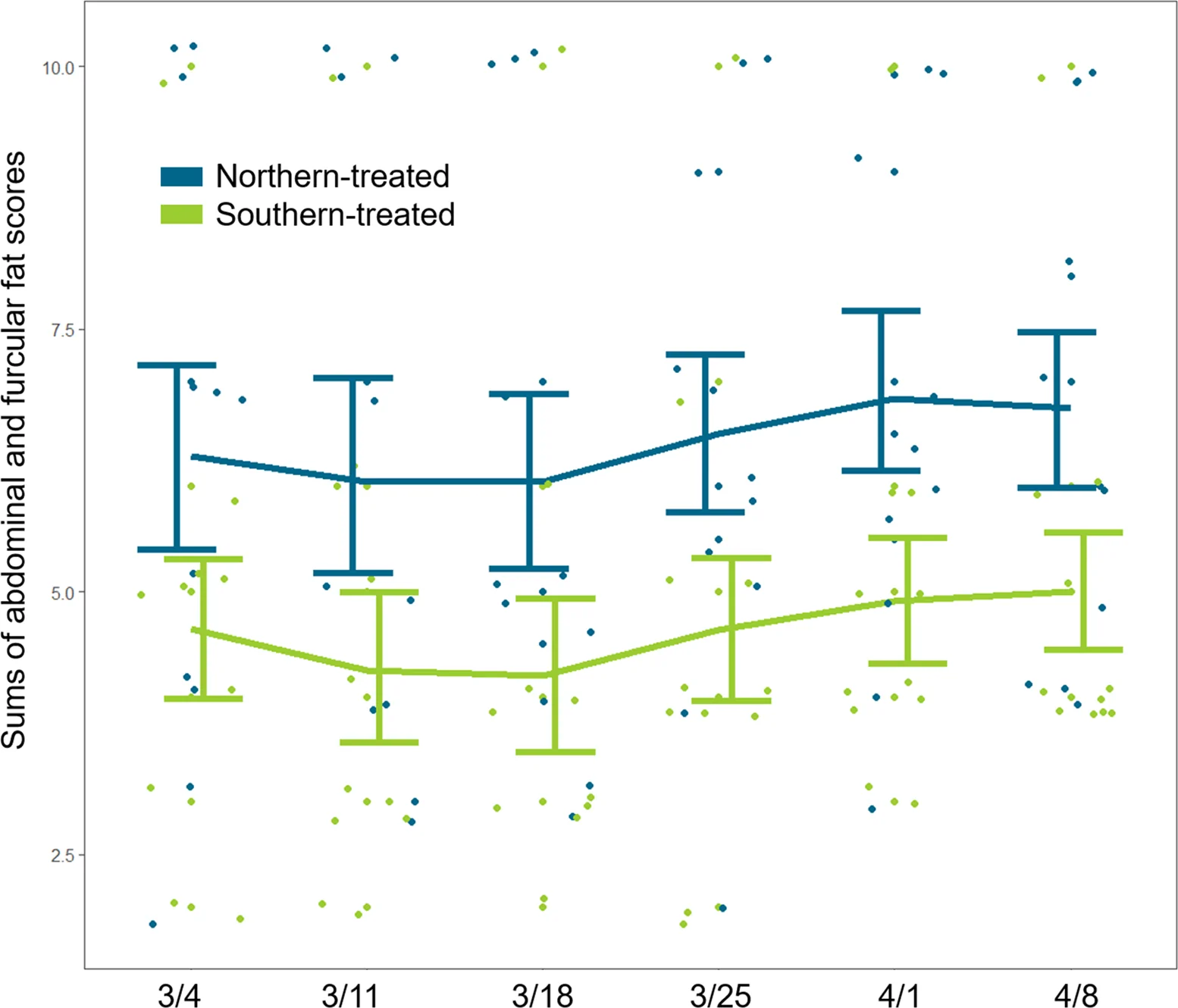
Sums of abdominal and furcular fat scores in females (mean ± s.e.m.). Sampling date and nest of origin influenced female fat deposition, but neither treatment nor its interaction with sampling date did. Note that while models were run with log-transformed values, raw values are graphed for ease of viewing.

## 4. Discussion

Experimentally manipulated developmental photoperiod altered the timing of reproductive development in male but not female dark-eyed juncos, with northern-treated males exhibiting lower circulating testosterone in response to GnRH in early- to mid-March, somewhat smaller CPs and somewhat higher fat scores. This is analogous to the findings in Fudickar *et al.* [24], in which wild-caught migrant male juncos from the northern extent of their breeding range, when held under common-garden conditions with resident males from southern overwintering grounds, similarly took longer in spring to match more southern males’ induced testosterone levels (the circulating testosterone concentrations in this study also closely aligned with those in Fudickar *et al.* [24]).

The experiment of Fudickar *et al.* [24] suggests local adaptation as the main means by which juncos time reproductive capacity for their breeding environments. However, because the birds were captured as adults, developmental plasticity in response to environment-specific cues (including photoperiod) cannot be ruled out [13,24]. The evidence presented here, together with that of Fudickar *et al.* [24], strongly suggests that at least some of the phenotypic divergence of reproductive timing in the dark-eyed junco's range may arise from the influence of developmental photoperiod.

Daylength treatment did not affect male fat deposition or CP, though both trended in the predicted directions. Northern-treated males’ CP volumes were consistently smaller compared with southern-treated males’ from mid-March through the end of sampling in early April. Northern-treated males also tended to score higher than southern-treated males in furcular and abdominal fat, as would be expected if exposure to northern photoperiods early in life activated preparation for a northward migration after their first winter at lower latitudes [37–39].

As noted, southern-treated males secreted more testosterone earlier in spring in response to GnRH injection than northern-treated males, indicating better-developed testes that were able to respond to hormone signalling for sperm production. Such elevated GnRH-induced testosterone levels corresponded with southern-treated males’ trend towards larger CPs, as androgens promote enlargement of the sperm storage organ to improve copulation [40].

Because the ability to transduce exogenous GnRH into reproductive readiness requires the coordination of multiple tissue types along the HPG axis, the northern-treated males’ relative inability to respond to GnRH could have several causes. One such mechanism for this reduced response could be a lack of or reduced GnRH receptor expression in the anterior pituitary [41,42]. Without binding to this G-protein-coupled receptor on the membranes of gonadotropic cells, GnRH cannot induce the phosphorylation pathway to produce LH and FSH [42]. However, we consider this an unlikely explanation for the northern-treated males’ lower testosterone levels, as previous work in juncos found that both free-living resident and migrant males consistently increased LH production following a GnRH injection [43,44].

Even if the pituitary produces sufficient LH and FSH, variation at the level of the gonad can contribute to temporal variation in the development of reproductive physiology. White-winged juncos *(J. h. aikeni)* in the Black Hills of South Dakota tend to breed later than the slate-coloured juncos in Virginia (approximately 10 May versus 16 April) [45], personal communication; [46], personal observation; [47]. Bergeon Burns *et al.* [43] reported that within individual males from these populations, testis mass positively correlated with their ability to secrete testosterone in response to GnRH injection. Moreover, *J. h. carolinensis* males from Virginia expressed more LH receptor mRNA at the testis in early spring compared with later-breeding white-winged junco males in South Dakota, despite the populations exhibiting nearly identical levels of GnRH-induced LH or LH-induced testosterone [43]. These findings indicate that gonadal development (via mass and gonadotrope receptor expression) could contribute to individual- and population-level differences in reproductive timing [43].

The greater reproductive capacity exhibited earlier in the year by males exposed to the southern developmental photoperiod treatment may stem from their experience of shorter days after hatching (simulating lower latitudes) compared with northern-treated males (which experienced longer days after hatching to mimic summers in Alaska). Such relatively shorter maximum postnatal daylengths could establish lower photoperiodic thresholds; that is, shorter minimum durations of daylight within the circadian cycle able to trigger the seasonal reproductive phenotype [4,11]. In mice, the daylengths experienced postnatally can influence circadian rhythms into adulthood [19]. Such resultant variation in circadian rhythms could underpin population-level variation in annual rhythms, explaining diversity in the timing of the onset of reproduction across latitudes [4,10,11,19,20].

Under this framework, the northern-treated males’ experience of longer photoperiods early in life would have set higher photoperiodic thresholds and the requirement of longer daylengths to initiate reproductive readiness in subsequent springs. Waiting for daylengths to surpass a given photoperiodic threshold to initiate reproduction enables individuals to coordinate the timing of energy expenditure—from GnRH secretion all the way to behaviour—with improved chances for offspring success and fitness according to environmental favourability [2,3].

Although the photoperiod the northern-treated males experienced as nestlings and fledglings appears to have influenced later responses to daylength, birds in this group were also exposed to daylengths mimicking an autumnal southward migration. Therefore, we are unable to clearly discern whether treatment groups’ differences arose from the photoperiods detected during a critical developmental window of sensitivity or the photoperiod changes mimicking migration (or its absence). One important caveat is that while we mimicked changes in photoperiod associated with southward migration, we could not wholly simulate a southward migration for northern-treated birds in captivity. Migration is an energetically intense, multisensory phenomenon as birds nocturnally travel distances as great as continents via electromagnetic and stellar signals, all while enduring substantial demands on body fat and muscular stores [48]. As we were unable to provide the full spectrum of migratory cues, our treatment modulation of only photoperiod may have blunted springtime differences in the treatments’ hormonal and morphological profiles.

In females, BP development and GnRH-induced testosterone did not detectably change in either group for the duration of the study. Sampling date but not treatment affected abdominal and furcular fat deposition, if not in an easily interpretable pattern: summed fat scores slightly dipped around mid-March before rising again. Northern-treated females trended towards higher fat scores, consistent with preparation for migration before reproduction [37–39]. The significant effect of nest of origin on fat deposition could implicate the role of shared genetics or very early environmental factors (since nestlings were brought into captivity their second day of life) in how birds respond to their first seasonal photoperiodic changes.

The lack of observable differences between treatments in females may stem from female birds simply becoming reproductive later in the season than males, sometimes by as much as 5 weeks [14,49,50]. For reference, our final sampling date was 8 April, while the median first egg date of resident juncos in Virginia was 5 May in 2015, with BPs appearing several days to weeks before laying [5,23]. As females invest relatively more energy per egg than males do per spermatozoon, they face stronger selective pressure to be more sensitive to proximate cues (e.g. rainfall, temperature, food availability, behaviour of the opposite sex) than males [51–53]. Females individually caged within indoor facilities may not receive the suite of input necessary to initiate reproduction in the wild. Such high specificity and required amount of cues may explain the observed difficulties of breeding wild-caught birds in captivity (reviewed in [54] and [55]). Studying females under more natural conditions that are more amenable to the initiation of a reproductive phenotype would enable investigation of how females integrate cues to time reproduction, including the potential role of photoperiod and developmental plasticity in this timing.

Holistically, the disparity between treatments in GnRH-induced testosterone in males suggests that developmental photoperiod indeed influences the timing of reproduction in male dark-eyed juncos. We acknowledge that many studies demonstrate a heritable component to the onset of reproduction ([10,14,17,56,57], to name only a few). If local adaptation is the primary driver of seasonal timing, then depending on the magnitude and speed of future changes in climate, the photoperiods birds experience when young might not bear substantial influence on reproductive timing. This could increase the risk of species decline as populations shift their ranges in response to environmental change and habitat availability, exacerbating phenological mismatch [58].

However, recent work suggests that reproductive rhythms attributed to heritability in bird populations can be largely explained by environmental similarity [59]. Furthermore, the problem remains of how highly genetically similar populations can exhibit the observed variation in the timing of breeding across latitudes [18,60,61]. Most probably, interindividual variation in the onset of reproduction is an emergent property of genetic and environmental (G × E) forces [18]. This interaction, combined with environmental erraticism that is proposed to increase with climate change, could exert a selective pressure for the maintenance of the genetic capacity of plasticity [18,62–64].

Plasticity in reproductive timing could be hopeful news in light of a warming and increasingly unpredictable climate. Should certain passerines face pockets of population decline, conspecifics immigrating from different latitudes (be this through natal dispersal or anthropogenic assistance) may be able to adopt the photoperiodic schedule of their new range and successfully integrate and breed with their new population [19,57,62]. Indeed, the work of Berthold, Pulido and associates has documented several passerine families’ capacities to adjust to novel overwintering latitudes and migratory schedules owing to both genetic and phenotypic variability [62,65–67]. Such sensitivity to developmental conditions may explain how individuals are able to disperse from their natal environment and successfully integrate into breeding populations at different latitudes [57,68].

Because the birds in our study were from the same population, we suggest the observed differences between treatments stem from plasticity in response to developmental photoperiod, which influenced the circadian system and its regulated responses [4,69]. Further research should explore the interplay of plasticity and genotypes in seasonal timing and potential sex-specific differences in plastic responses to developmental photoperiod.

## Supplementary Material



## Data Availability

Data and code are accessible in Zenodo [70]. Supplementary material is available online [71].

## References

[1] Goldman BD . 2001 Mammalian photoperiodic system: formal properties and neuroendocrine mechanisms of photoperiodic time measurement. J. Biol. Rhythms 16, 283–301. (doi:10.1177/074873001129001980) 11506375

[2] Lack D . 1950 The breeding seasons of European birds. Ibis 92, 288–316. (doi:10.1111/j.1474-919x.1950.tb01753.x)

[3] Bronson FH . 1985 Mammalian reproduction: an ecological perspective. Biol. Reprod. 32, 1–26. (doi:10.1095/biolreprod32.1.1) 3882162

[4] Dawson A , King VM, Bentley GE, Ball GF. 2001 Photoperiodic control of seasonality in birds. J. Biol. Rhythms 16, 365–380. (doi:10.1177/074873001129002079) 11506381

[5] Kimmitt AA , Becker DJ, Diller SN, Gerlach NM, Rosvall KA, Ketterson ED. 2022 Plasticity in female timing may explain earlier breeding in a North American songbird. J. Anim. Ecol. 91, 1988–1998. (doi:10.1111/1365-2656.13772) 35819093

[6] Wingfield JC , Hahn TP, Levin R, Honey P. 1992 Environmental predictability and control of gonadal cycles in birds. J. Exp. Zool. 261, 214–231. (doi:10.1002/jez.1402610212)

[7] Fox SI , Rompolski K. 2019 Human physiology, 15th edn. New York City, NY: McGraw-Hill Education.

[8] Edge CB , Rollinson N, Brooks RJ, Congdon JD, Iverson JB, Janzen FJ, Litzgus JD. 2017 Phenotypic plasticity of nest timing in a post-glacial landscape: how do reptiles adapt to seasonal time constraints? Ecology 98, 512–524. (doi:10.1002/ecy.1665) 27870008

[9] Richter-Boix A , Llorente GA, Montori A. 2006 Breeding phenology of an amphibian community in a Mediterranean area. Amphib.-Reptil. 27, 549–559. (doi:10.1163/156853806778877149)

[10] Silverin B , Massa R, Stokkan KA. 1993 Photoperiodic adaptation to breeding at different latitudes in great tits. Gen. Comp. Endocrinol. 90, 14–22. (doi:10.1006/gcen.1993.1055) 8504918

[11] Singh D , Reed SM, Kimmitt AA, Alford KA, Stricker CA, Polly PD, Ketterson ED. 2021 Breeding at higher latitude is associated with higher photoperiodic threshold and delayed reproductive development in a songbird. Horm. Behav. 128, 104 907. (doi:10.1016/j.yhbeh.2020.104907) 33259797

[12] Wanamaker SM , Singh D, Byrd AJ, Smiley TM, Ketterson ED. 2020 Local adaptation from afar: migratory bird populations diverge in the initiation of reproductive timing while wintering in sympatry. Biol. Lett. 16, 20200493. (doi:10.1098/rsbl.2020.0493) PMC7655480 33023381

[13] Ketterson ED , Greives TJ. 2025 Mechanisms matching timing to resources: comparisons of closely related seasonally sympatric, migratory and non-migratory populations. J. Avian Biol. 2025, e03380. (doi:10.1002/jav.03380)

[14] Caro SP , Charmantier A, Lambrechts MM, Blondel J, Balthazart J, Williams TD. 2009 Local adaptation of timing of reproduction: females are in the driver's seat. Funct. Ecol. 23, 172–179. (doi:10.1111/j.1365-2435.2008.01486.x)

[15] Lambrechts MM , Dias PC. 1993 Differences in the onset of laying between island and mainland Mediterranean blue tits *Parus caeruleus*: phenotypic plasticity or genetic differences? Ibis 135, 451–455. (doi:10.1111/j.1474-919x.1993.tb02118.x)

[16] Liedvogel M , Szulkin M, Knowles SC, Wood MJ, Sheldon BC. 2009 Phenotypic correlates of *clock* gene variation in a wild blue tit population: evidence for a role in seasonal timing of reproduction. Mol. Ecol. 18, 2444–2456. (doi:10.1111/j.1365-294x.2009.04204.x) 19457200

[17] Noordwijk AJV , Van Balen JH, Scharloo W. 1980 Heritability of ecologically important traits in the great tit. Ardea 68, 193–203. (doi:10.5253/arde.v68.p193)

[18] Hahn TP , MacDougall-Shackleton SA. 2008 Adaptive specialization, conditional plasticity and phylogenetic history in the reproductive cue response systems of birds. Phil. Trans. R. Soc. Lond. Ser. B 363, 267–286. (doi:10.1098/rstb.2007.2139) PMC2606750 17686737

[19] Ciarleglio CM , Axley JC, Strauss BR, Gamble KL, McMahon DG. 2010 Perinatal photoperiod imprints the circadian clock. Nat. Neurosci. 14, 25–27. (doi:10.1038/nn.2699) PMC3058292 21131951

[20] Follett BK . 1973 Circadian rhythms and photoperiodic time measurement in birds. J. Reprod. Fertil. 19, 5–18. https://pubmed.ncbi.nlm.nih.gov/4594248/. 4594248

[21] MacDougall-Shackleton SA , Stevenson TJ, Watts HE, Pereyra ME, Hahn TP. 2009 The evolution of photoperiod response systems and seasonal GnRH plasticity in birds. Integr. Comp. Biol. 49, 580–589. (doi:10.1093/icb/icp048) 21665842

[22] Bauer CM , Fudickar AM, Anderson-Buckingham S, Abolins-Abols M, Atwell JW, Ketterson ED, Greives TJ. 2018 Seasonally sympatric but allochronic: differential expression of hypothalamic genes in a songbird during gonadal development. Proc. R. Soc. B 285, 20 181 735. (doi:10.1098/rspb.2018.1735) PMC6234895 30355713

[23] Nolan V , Ketterson ED, Cristol DA, Rogers CM, Clotfelter ED, Titus RC, Schoech SJ, Snajdr E. 2002Dark-eyed junco *(Junco hyemalis)*. In The birds of North America (eds AF Poole, FB Gill). Ithaca, NY: Cornell Lab Ornithology.

[24] Fudickar AM , Greives TJ, Atwell JW, Stricker CA, Ketterson ED. 2016 Reproductive allochrony in seasonally sympatric populations maintained by differential response to photoperiod: implications for population divergence and response to climate change. Am. Nat. 187, 436–446. (doi:10.1086/685296) 27028072

[25] Ketterson ED , Fudickar AM, Atwell JW, Greives TJ. 2015 Seasonal timing and population divergence: when to breed, when to migrate. Curr. Opin. Behav. Sci. 6, 50–58. (doi:10.1016/j.cobeha.2015.09.001)

[26] Adam I , Scharff C, Honarmand M. 2014 Who is who?Non-invasive methods to individually sex and mark altricial chicks. J. Vis. Exp. 87, e51429. (doi:10.3791/51429) PMC4207129 24893585

[27] Byrd AJ , Mullins CS, Becker DJ, Fudickar AM. 2023 Nestling growth rate and food consumption increases under experimentally prolonged daylength in a New World sparrow. J. Avian Biol. 2023, e03113. (doi:10.1111/jav.03113)

[28] Jawor JM , McGlothlin JW, Casto JM, Greives TJ, Snajdr EA, Bentley GE, Ketterson ED. 2006 Seasonal and individual variation in response to GnRH challenge in male dark-eyed juncos *(Junco hyemalis)*. Gen. Comp. Endocrinol. 149, 182–189. (doi:10.1016/j.ygcen.2006.05.013) 16814785

[29] Nolan V , Ketterson ED. 1983 An analysis of body mass, wing length, and visible fat deposits of dark-eyed juncos wintering at different latitudes. Wilson Bull. 95, 603–620. https://www.jstor.org/stable/4161832.

[30] Hoi H . 1998 Individual and temporal variation in cloacal protuberance size of male bearded tits *(Panurus biarmicus)*. Auk 115, 964–969. (doi:10.2307/4089514)

[31] Graham JL , Needham KB, Bertucci EM, Pearson AA, Bauer CM, Greives TJ. 2019 Onset of daily activity in a female songbird is related to peak-induced estradiol levels. Integr. Comp. Biol. 59, 1059–1067. (doi:10.1093/icb/icz112) 31236557

[32] Kimmitt AA , Webb AL, Greives TJ, Ketterson ED. 2020 Migrant and resident female songbirds differ in gonadal response to upstream stimulation during seasonal sympatry. Gen. Comp. Endocrinol. 293, 113469. (doi:10.1016/j.ygcen.2020.113469) 32220572

[33] Rosvall KA , Bergeon Burns CM, Hahn TP, Ketterson ED. 2013 Sources of variation in HPG axis reactivity and individually consistent elevation of sex steroids in a female songbird. Gen. Comp. Endocrinol. 194, 230–239. (doi:10.1016/j.ygcen.2013.09.015) PMC3852689 24090613

[34] Kuznetsova A , Brockhoff PB, Christensen RH. 2017 lmerTest package: tests in linear mixed effects models. J. Stat. Soft. 82, 1–26. (doi:10.18637/jss.v082.i13)

[35] Bates D , Mächler M, Bolker B, Walker S. 2015 Fitting linear mixed-effects models using lme4. J. Stat. Soft. 67, 148. (doi:10.18637/jss.v067.i01)

[36] Lenth R . 2024 *emmeans*: Estimated Marginal Means, aka Least-Squares Means. R package version 1.10.4. https://CRAN.R-project.org/package=emmeans.

[37] Martin T . 1987 Food as a limit on breeding birds: a life-history perspective. Annu. Rev. Ecol. Syst. 18, 453–487. (doi:10.1146/annurev.ecolsys.18.1.453)

[38] Sandberg R , Moore FR. 1996 Fat stores and arrival on the breeding grounds: reproductive consequences for passerine migrants. Oikos 77, 577. (doi:10.2307/3545949)

[39] Schwabl H , Kriner E. 1991 Territorial aggression and song of male European robins (Erithacus Rubecula) in autumn and spring: Effects of antiandrogen treatment. Horm. Behav. 25, 180–194. 10.1016/0018-506x(91)90049-n 2066079

[40] Zera AJ , Harshman LG. 2001 The physiology of life history trade-offs in animals. Annu. Rev. Ecol. Syst. 32, 95–126. (doi:10.1146/annurev.ecolsys.32.081501.114006)

[41] Bédécarrats GY , Shimizu M, Guémené D. 2006 Gonadotropin releasing hormones and their receptors in avian species. J. Poult. Sci. 43, 199–214. (doi:10.2141/jpsa.43.199)

[42] Rispoli LA , Nett TM. 2005 Pituitary gonadotropin-releasing hormone (GnRH) receptor: structure, distribution and regulation of expression. Anim. Reprod. Sci. 88, 57–74. (doi:10.1016/j.anireprosci.2005.05.004) 15993012

[43] Kimmitt AA *et al*. 2023 Genetic evidence for widespread population size expansion in North American boreal birds prior to the last glacial maximum. Proc. R. Soc. B. 290, 20221334. (doi:10.1098/rspb.2022.1334) PMC9874272 36695033

[44] Berthold PE . 2001 Bird migration: A general survey. Oxford, England: Oxford University Press.

[45] Bergeon Burns CM , Rosvall KA, Hahn TP, Demas GE, Ketterson ED. 2014 Examining sources of variation in HPG axis function among individuals and populations of the dark-eyed junco. Horm. Behav. 65, 179–187. (doi:10.1016/j.yhbeh.2013.10.006) PMC3944345 24140626

[46] Greives TJ , Kingma SA, Beltrami G, Hau M. 2016 Melatonin delays clutch initiation in a wild songbird. Biol. Lett. 8, 330–332. (doi:10.1098/rsbl.2011.1100) PMC3367749 22171024

[47] Greives TJ . 2025 Personal communication.

[48] Saline CG . 2024 Personal observation.

[49] Dawson A . 2007 Control of the annual cycle in birds: endocrine constraints and plasticity in response to ecological variability. Phil. Trans. R. Soc. Lond. Ser. B 363, 1621–1633. (doi:10.1098/rstb.2007.0004) PMC2606722 18048294

[50] Kimmitt AA . 2020 Females as the gatekeepers to seasonal breeding: what we can learn by studying reproductive mechanisms in both sexes. Integr. Comp. Biol. 60, 703–711. (doi:10.1093/icb/icaa095) 32617554

[51] Ball GF , Ketterson ED. 2007 Sex differences in the response to environmental cues regulating seasonal reproduction in birds. Phil. Trans. R. Soc. Lond. Ser. B 363, 231–246. (doi:10.1098/rstb.2007.2137) PMC2606748 17638693

[52] Watts HE , Robart AR, Hahn TP, Gomulkiewicz R. 2022 Environmental cue integration and phenology in a changing world. Integr. Comp. Bio. 62, 972–979. (doi:10.1093/icb/icac032) 35551401

[53] Wingfield JC , Hahn TP, Wada M, Schoech SJ. 1997 Effects of day length and temperature on gonadal development, body mass, and fat depots in white-crowned sparrows, *Zonotrichia Leucophrys pugetensis.* Gen. Comp. Endocrinol. 107, 44–62. (doi:10.1006/gcen.1997.6894) 9208305

[54] Griffith SC *et al.* 2016 Variation in reproductive success across captive populations: methodological differences, potential biases and opportunities. Ethology 123, 1–29. (doi:10.1111/eth.12576)

[55] Mason GJ . 2010 Species differences in responses to captivity: stress, welfare and the comparative method. Trends Ecol. Evol. 25, 713–721. (doi:10.1016/j.tree.2010.08.011) 20952089

[56] Heideman PD , Bruno TA, Singley JW, Smedley JV. 1999 Genetic variation in photoperiodism in *Peromyscus leucopus*: geographic variation in an alternative life-history strategy. J. Mammal. 80, 1232–1242. (doi:10.2307/1383173)

[57] Lamers KP , Nilsson J-Å, Nicolaus M, Both C. 2023 Adaptation to climate change through dispersal and inherited timing in an avian migrant. Nat. Ecol. Evol. 7, 1869–1877. (doi:10.1038/s41559-023-02191-w) 37710043

[58] Beever EA *et al.* 2015 Improving conservation outcomes with a new paradigm for understanding species’ fundamental and realized adaptive capacity. Conserv. Lett. 9, 131–137. (doi:10.1111/conl.12190)

[59] Jones CV , Regan CE, Cole EF, Firth JA, Sheldon BC. 2025 Shared environmental similarity between relatives influences heritability of reproductive timing in wild great tits. Evolution 79, 220–231. (doi:10.1093/evolut/qpae155) 39497526

[60] Milá B , McCormack JE, Castañeda G, Wayne RK, Smith TB. 2007 Recent postglacial range expansion drives the rapid diversification of a songbird lineage in the genus *Junco*. Proc. R. Soc. B 274, 2653–2660. (doi:10.1098/rspb.2007.0852) PMC2279216 17725978

[61] Schoech SJ , Hahn TP. 2007 Food supplementation and timing of reproduction: does the responsiveness to supplementary information vary with latitude? J. Ornithol. 148, 625–632. (doi:10.1007/s10336-007-0177-6)

[62] Pulido F , Berthold P. 2004 Microevolutionary response to climatic change. Adv. Ecol. Res. 35, 151–183. (doi:10.1016/S0065-2504(04)35008-7)

[63] Scheiner SM . 1993 Genetics and evolution of phenotypic plasticity. Ann. Rev. Ecol. Syst. 24, 35–68. (doi:10.1146/annurev.ecolsys.24.1.35)

[64] Wigley TM . 2009 The effect of changing climate on the frequency of absolute extreme events. Clim. Change 97, 67–76. (doi:10.1007/s10584-009-9654-7)

[65] Berthold P , Pulido F. 1994 Heritability of migratory activity in a natural bird population. Proc. R. Soc. B 257, 311–315. (doi:10.1098/rspb.1994.0131)

[66] Coppack T , Pulido F, Czisch M, Auer DP, Berthold P. 2003 Photoperiodic response may facilitate adaptation to climatic change in long-distance migratory birds. Proc. R. Soc. B 270, S43–S46. (doi:10.1098/rsbl.2003.0005) PMC1698020 12952632

[67] Pulido F , Berthold P, Mohr G, Querner U. 2001 Heritability of the timing of autumn migration in a natural bird population. Proc. R. Soc. B 268, 953–959. (doi:10.1098/rspb.2001.1602) PMC1088693 11370969

[68] Studds CE , Kyser TK, Marra PP. 2008 Natal dispersal driven by environmental conditions interacting across the annual cycle of a migratory songbird. Proc. Natl Acad. Sci. USA 105, 2929–2933. (doi:10.1073/pnas.0710732105) PMC2268562 18287030

[69] Nicholls TJ , Goldsmith AR, Follett BK, Dawson A. 1987 The photoperiodic responses of European starlings *(Sturnus vulgaris)*. Adapt. Clim. Change 3, 23–29. (doi:10.1159/000413971)

[70] Saline CG , Fudicker A, Byrd AJ, Ketterson ED, Greives TJ. 2026 salinedi0n/Junco-Dev-Photoperiod-PRSB-2026: Junco-Dev-Photoperiod-PRSB-2026 Zenodo Release [Dataset]. Zenodo. (doi:10.5281/zenodo.21706578)

[71] Saline CG , Fudicker A, Byrd AJ, Ketterson ED, Greives TJ. 2026 Supplementary material from: Developmental photoperiod alters the onset and capacity for reproduction in male dark-eyed juncos (*Junco hyemalis*). Figshare. (doi:10.6084/m9.figshare.c.8664635) PMC13678725 42869484

