# Supplementary material for: Developmental photoperiod alters the onset and capacity for reproduction in male dark-eyed juncos (Junco hyemalis)
Source: Proc Biol Sci. 2026 Oct 14;293(2080):20261696. doi: 10.1098/rspb.2026.1696 (PMC13678725; doi:10.1098/rspb.2026.1696)
Supplement: Supplementary file 1 [file rspb.2026.1696.zip › rspb.2026.1696/rspb20261696_si_015.docx]

Electronic supplementary material key for:

Saline CG, Fudickar AM, Byrd AJ, Ketterson ED, Greives TJ. 2026 Developmental photoperiod alters the onset and capacity for reproduction in male Dark-eyed Juncos *(Junco hyemalis). Proc. R. Soc. B.* http://dx.doi.org/10.1098/rspb.2026.1696

“Please provide a descriptive title (not the file name) and a short description for each separate file. These will be used online and must provide enough information for a reader to know what the supplement is before opening the file. If you have more than 5 files, please upload these titles and captions as a document along with your manuscript files.”

ESM file 1

Title: README

Legend/Caption: Summary of study, files, software, and code.

ESM file 2

Title: CODE

Legend/Caption: Annotated R script to conduct statistical analyses and produce graphs.

ESM file 3

Title: Male GnRH-induced testosterone

Legend/Caption: Males’ GnRH-induced testosterone values by treatment and sampling week.

ESM file 4

Title: Male fat

Legend/Caption: Males’ furcular and abdominal fat scores by treatment and sampling week.

ESM file 5

Title: Cloacal protuberance (CP)

Legend/Caption: Males’ CP volumes by treatment and sampling week.

ESM file 6

Title: Female GnRH-induced testosterone

Legend/Caption: Females’ GnRH-induced testosterone values by treatment and sampling week.

ESM file 7

Title: Female fat

Legend/Caption: Females’ furcular and abdominal fat scores by treatment and sampling week.

ESM file 8

Title: Photoperiod schematic

Legend/Caption: Hours of daylight each treatment received by Julian date.
